# The perception of plastic surgery during the COVID-19 pandemic—an analysis of online search patterns on a medical information platform

**DOI:** 10.1057/s41599-023-01625-x

**Published:** 2023-03-24

**Authors:** Sebastian P. Nischwitz, Janis Jung, Hanna Luze, Daniel Popp, Robert Zrim, Thomas Rappl, Lars-Peter Kamolz, Stephan Spendel

**Affiliations:** 1 Division of Plastic, Aesthetic and Reconstructive Surgery, Department of Surgery, Medical University of Graz, Graz, Austria; 2 MOOCI GmbH, Vienna, Austria; 3 International University of Monaco, Rue Hubert Clerissi, Monaco; 4 COREMED—Cooperative Centre for Regenerative Medicine, JOANNEUM RESEARCH Forschungsgesellschaft mbH, Graz, Austria

**Keywords:** Medical humanities, Science, technology and society

## Abstract

In 2020, the COVID-19 pandemic impacted global life and transitioned economies and societal perceptions of life as we knew it. Professional and social life mostly ground to a nadir during the first lockdown in Europe in March. As a consequence, measures aimed at preventing the spread of the virus were established in medical facilities also and elective plastic surgery procedures were temporarily suspended in our clinic and others. A majority of the population, including those potentially contemplating plastic surgery procedures, spent most of their time at home with ample time available to research information about surgical procedures and other topics online. This investigation analyzes the relevance of plastic surgery during the pandemic on the basis of online search behavior patterns. Online traffic data from the online platform http://www.mooci.org were extracted using Google Analytics over a period of 6 months. The parameters analyzed were: pageviews, session duration, and bounce rate. Additionally, differentiation by areas of interest has been obtained. The data were compared and analyzed before and after the beginning of the first hard lockdown in Austria, Germany, and Switzerland. There were no significant differences in regard to pageviews and session duration when comparing time points before and after the beginning of the hard lockdown. The bounce rate exhibited a significant decrease after the beginning of the lockdown, implying a more conscious search for information and greater absorption and retention. There was no difference that could conclusively be attributed to the pandemic in terms of specific areas of interest researched. Society’s demand for information about plastic-surgical procedures continues to be steadily prevalent—despite, or even in particular, during a global pandemic. Providing reliable and readily available information about plastic surgery procedures is an important component of a functioning doctor–patient relationship and informed consent. This information may reflect society’s increased interest in plastic surgery during the pandemic, or be simply reflective of more spare time at hand to allow for such research. Further studies should investigate the relevance of elective procedures over the entire course of the pandemic.

## Introduction

The first months of 2020 were characterized by the emerging COVID-19 pandemic. Following its classification as a pandemic by the WHO on March 11, 2020, many European countries introduced measures to delay or prevent the spread of the novel Coronavirus. Such measures were enforced in Austria and Switzerland on March 16, 2020 (Federal Government of Austria, 2020), and in Germany on March 22, 2020 (Auswärtiges Amt der Bundesregierung Deutschland, 2020) and comprised different curfews depending on the region, as well as mandatory face masks covering mouth and nose in public. Consequently, the measures brought public and social life to a halt, but also the professional life of many individuals, thus heavily affecting the general public, not last by inciting or uncovering insecurities and existential fear (Wang et al., 2020).

The medical and healthcare systems were forced to scale back their daily clinical routine to a baseline to preserve capacities for the anticipated influx of COVID-19 patients and emergency presentations. As a consequence, elective surgeries had to be postponed until further notice (Armstrong et al., 2020; Nischwitz et al., 2020).

Uncertainty concerning the potential health consequences of the pandemic was omnipresent. Plastic surgery, which is publicly often only perceived as a*esthetic surgery*, was affected by these uncertainties as well.

Patients contemplating plastic surgery procedures were not able to gather information at their doctor’s office and increasingly turned to online research to retrieve information about doctors, treatment options, pros and cons, and other relevant aspects about plastic surgery (Nischwitz et al., 2021). While there is a lot of publicly available but popularized information about plastic surgery, high-quality content offered in a serious manner is relatively rare. Quality and information platforms, such as MOOCI GmbH (MOOCI. Plastische Chirurgie, Dermatologie & Zahnmedizin), SEOrello GmbH (Ärzteportal—Arztportal—Arztsuche in Ihrer Region bei med-doc24) or jameda GmbH (jameda—Arzttermine online buchen & Bewertungen lesen) are valuable platforms for potential patients in German-speaking countries.

Due to the pandemic, drastically impacting almost everybody’s lives, we assumed changes regarding the online search pattern behavior of patients seeking information about plastic surgery procedures. Many people, including a large number of potential patients, spent most of their days at home or in the home office, leading to the assumption of a large(r) share of online time and contemplation of health and medical issues. We anticipated that the majority of the available time spent online would mostly focus on general medical and health topics (focusing on COVID-19), with unchanged or even decreased interest in information about plastic surgery procedures. Furthermore, we assumed that the pandemic led to more focused search patterns and longer time spent on specific websites. The aim of this study was to explore and emphasize the increasing importance of valid and serious medical information about plastic surgery online by assessing the above-mentioned aspects in a quali- and quantitative manner.

## Methods

The study was approved by the competent ethics committee (EK: 32-532 ex 19/20). All data was gathered completely anonymously.

### Study course

The online traffic data of *MOOCI GmbH* website have been extracted and analyzed using *Google Analytics*. Anonymous data had been provided free-of-charge by *MOOCI GmbH*, a platform providing professionally validated information to potential patients. *Google Analytics* is provided by *Google Ireland Limited*, and tracks the behavior of visitors once implemented on a website (Analytics Tools & Solutions for Your Business—Google Analytics). Data analysis has been limited to plastic surgery topics. *Google Analytics* data obtained between December 2019 and May 2020 has been subtracted and analyzed; totaling 26 weeks and comprising the beginning of the first lockdown in Germany (March 22, 2020), Austria, and Switzerland (March 16, 2020).

#### Structure of the time frame

The investigation period was divided into two parts: prior to—and during the first hard lockdown. Ultimately, there was a period of 16 weeks for Germany and 15 weeks for Austria and Switzerland prior to the lockdown, and 10 and 11 weeks of lockdown, respectively, due to the later lockdown in Germany. Hard lockdown is defined as the first initiation of a lockdown with a total halt of social life as compared to subsequent following soft lockdowns, during which only parts of social life were affected.

Data were summarized into three different periods and analyzed separately: *4 weeks* (four weeks prior to- vs. during the lockdown), *8 weeks* (*eight prior to- vs. eight during-*), and *total* (16 vs. 10 weeks for Germany, and 15 vs. 11 weeks for Austria and Switzerland, including prior to- and lockdown periods).

#### Variables

The following variables have been extracted and investigated:

*Page views:* The absolute number of visitors to a particular page on the website.

*Session duration:* The duration of a pageview indicated in seconds. Visits that ended after the first view on the website without further interaction (no further click) are not captured in this variable.

*Bounce rate:* The share of page views that ended without further interaction and led to the immediate closure of the webpage. Page views concluding in bounces do not contribute to the session duration.

#### Areas of interest

This investigation has further differentiated the areas of interest that are offered on the platform. Each topic has been allocated to one of the following areas of interest: Hand-/foot-surgery (I), Facial surgery (II), Body contouring (III), Intimate surgery (IV), and breast surgery (V).

### Statistical analysis

Data has been analyzed using descriptive statistics. To describe inferential statistics, a Student’s *t*-test has been performed for the comparison of two groups (prior/post). Before the Student’s *t*-test, data were tested for normal distribution using the Kolmogorov–Smirnov test (all yielded normal distribution). Significance has been set to *p* < 0.05.

## Results

This study investigated the online search pattern behavior of potential plastic surgery patients concerning information about plastic surgery procedures using data from the online platform of *MOOCI GmbH* in Germany, Austria, and Switzerland during the first hard lockdown during the COVID-19 pandemic in 2020.

The results have been analyzed for three different periods (four weeks prior to-/after, eight weeks prior to-/after, total) to prevent potential bias by a random choice of a specific period. The respective periods will be referred to as “4 weeks”, “8 weeks”, and “Total” throughout the manuscript. For the parameters *page views*, *session duration*, and *bounce rate* no significant differences were seen between the three countries Germany, Austria and Switzerland; therefore only pooled results are reported.

### Page views

Page views reflect the absolute number of visits on the particular page on the website.

#### 4 weeks

On average, there have been 48,715 page-views per week prior to the lockdown. During the lockdown, the weekly views dropped to 43,795. A tendency towards fewer page views can be seen, yet not to the level of statistical significance (*p* = 0.062).

#### 8 weeks

On average, there have been 44,854 page-views per week prior to the lockdown. During the lockdown, that metric dropped to 40,473 views per week. A tendency towards fewer page views can be seen, yet not to the level of statistical significance (*p* = 0.154).

#### Total

On average, there have been 39,344 page views per week during the studied period prior to the hard lockdown. During the lockdown, it has been 40,241 per week. A slight increase in page views was observed without reaching statistical significance (*p* = 0.743).

**Fig. 1:**
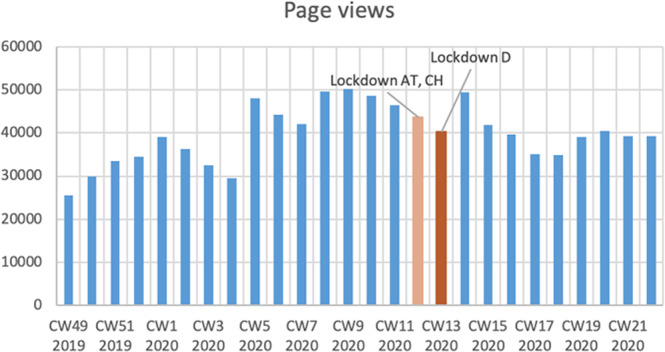
Course of absolute page views over time (average per week). A decline in page views at the beginning of the lockdown can be seen, which is quickly relativized. CW = Calendar week, AT = Austria, CH = Switzerland, D = Germany.

An overview of the page views is displayed in Fig. 1: The course of page views shows an undulating course with a tendency towards fewer page views immediately prior to the lockdown. Right after the beginning of the lockdown, this tendency is regressive and the number is rather stagnating. Over the course of the entire period investigated, a rising tendency can be seen.

### Session duration

The session duration indicates the average time in seconds a visitor remained on the website.

#### 4 weeks

A session lasted on average 137.41 s prior to the lockdown. During the lockdown, it lasted 133.43 s on average. The difference is not significant (*p* = 0.103).

#### 8 weeks

A session lasted on average 137.94 s prior to the lockdown. During the lockdown, it lasted 135.22 s on average. The difference is not significant (*p* = 0.060).

#### Total

A session lasted on average 140.04 s prior to the lockdown. During the lockdown, it lasted 134.57 s on average. The difference is not significant (*p* = 0.055).

**Fig. 2:**
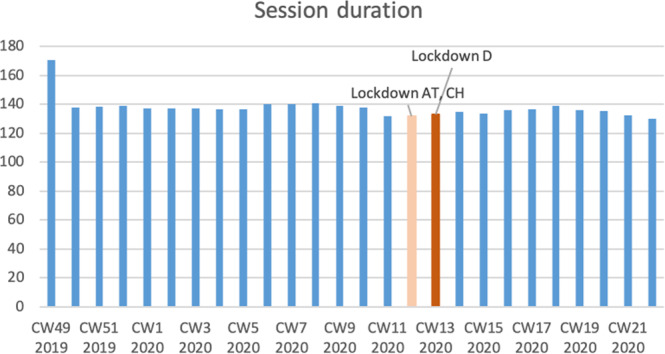
Course of the session duration over time (average per week). A more or less constant duration can be seen, that is not substantially influenced by the lockdown (Austria (AT), Switzerland (CH) = pink; Germany (D) = orange). CW = Calendar week.

Figure 2 displays an overview of the session duration: A more or less stable course is shown without substantial amplitudes.

### Bounce rate

The rate of all visits that ended without additional interaction on the website is referred to as the bounce rate. Visits ending in a bounce do not contribute to the session duration (see above).

#### 4 weeks

The bounce rate was 19.25% prior to the lockdown. During the lockdown, it decreased to 13.75. The difference is statistically significant (*p* = 0.048).

#### 8 weeks

The bounce rate was 19.63% prior to the lockdown. During the lockdown, it decreased to 13.63%. The difference is statistically highly significant (*p* < 0.001).

#### Total

The bounce rate was 19.53% prior to the lockdown. During the lockdown, it decreased to 14.45%. The difference is statistically highly significant (*p* < 0.001).

**Fig. 3:**
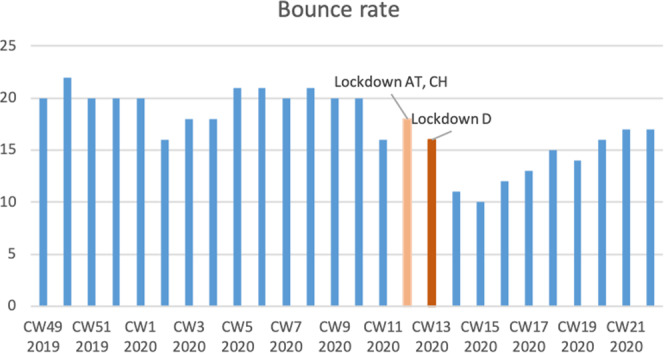
Bounce rate during the investigation (average per week). A significant decrease can be seen after the beginning of the lockdown (Austria (AT), Switzerland (CH) = pink; Germany (D) = orange). CW = Calendar week.

Figure 3 displays an overview of the bounce rate: A substantial decrease in the bounce rate can be seen immediately after the beginning of the lockdown, with subsequent slight increases.

### Areas of interest

This section describes the relative share of page views and session durations of the different areas of interest (see above).

#### 4 weeks

The results of the areas of interest are displayed in Table 1. There are no significant differences prior to- vs. during the lockdown neither in regards to page views nor session duration.

**Table 1:** Share of page views (above) and session duration (below) of the respective areas of interest 4 weeks prior- and after the beginning of the lockdown (Germany, Austria, and Switzerland).

	I	II	III	IV	V	Total
*Share of page views*						
Prior-	32.45%	25.49%	14.65%	13.90%	13.50%	100%
During-	31.95%	25.42%	14.79%	14.20%	13.64%	100%
*p*	0.163	0.804	0.511	0.272	0.567	
*Share of session duration*						
Prior-	20.09%	19.87%	20.07%	20.04%	19.93%	100%
During-	20.02%	19.91%	19.95%	20.07%	20.05%	100%
*p*	0.470	0.758	0.385	0.854	0.493	

I = Hand-/foot-surgery, II = Facial surgery, III = Body contouring, IV = Intimate surgery, V = Breast surgery, *p* = *p*-value.

#### 8 weeks

The results of the 8-week period are summarized in Table 2. A significant increase in the share of page views of intimate surgery topics is apparent. No further statistical differences were observed.

**Table 2:** Share of page views (above) and session duration (below) of the respective issue areas 8 weeks prior- and after the beginning of the lockdown (Germany, Austria and Switzerland).

	I	II	III	IV	V	Total
*Share of page views*						
Prior-	32.55%	25.45%	14.80%	13.69%	13.50%	100%
During-	32.31%	25.35%	14.72%	14.23%	13.39%	100%
*p*	0.501	0.688	0.624	0.006*	0.477	
*Share of session duration*						
Prior-	20.07%	19.95%	20.07%	20.01%	19.90%	100%
During-	20.15%	20.02%	19.84%	20.12%	19.87%	100%
*p*	0.287	0.427	0.064	0.322	0.821	

I = Hand-/foot-surgery, II = Facial surgery, III = Body contouring, IV = Intimate surgery, V = Breast surgery, *p* = *p*-value.

*Statistically significant.

#### Total

**Fig. 4:**
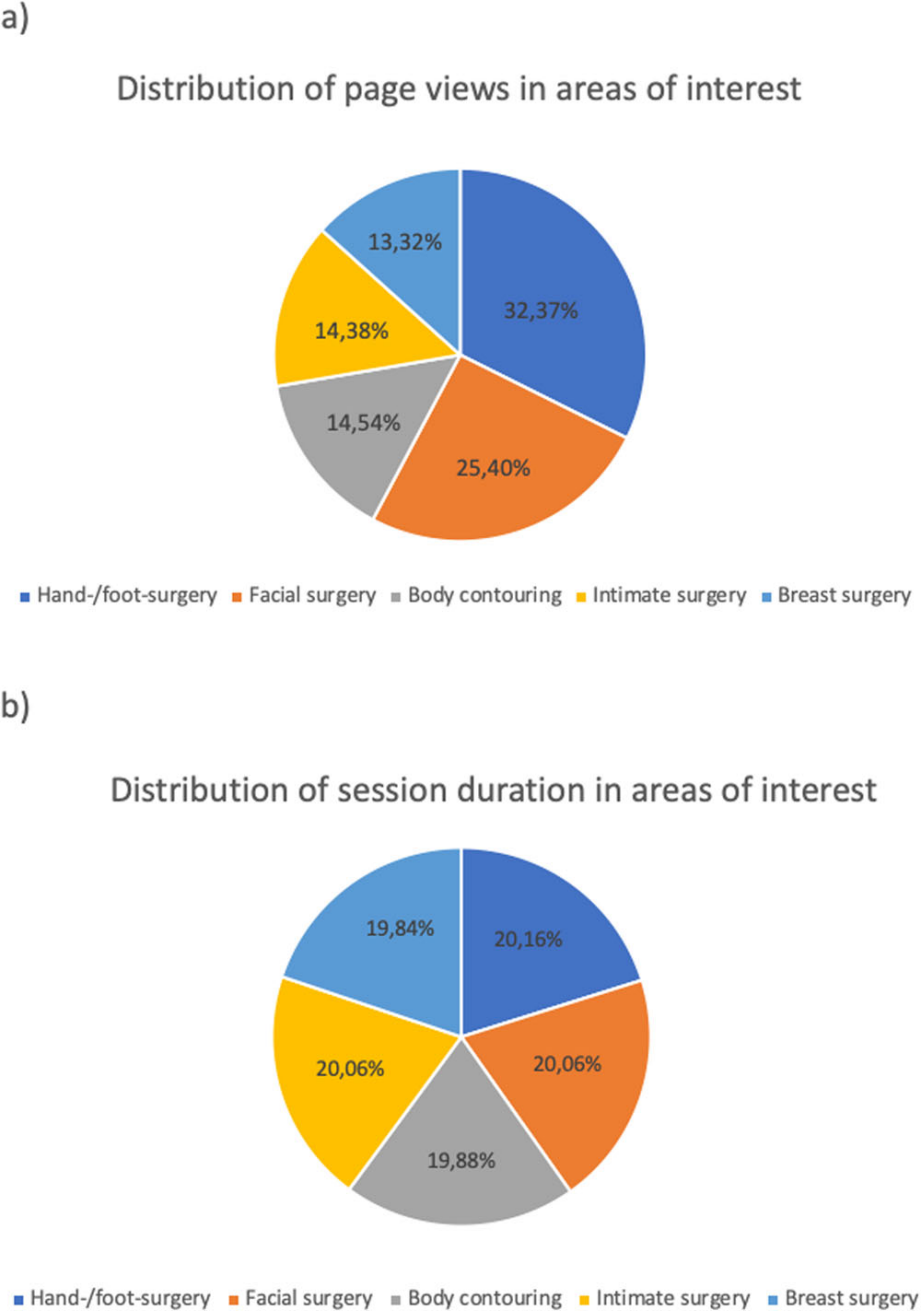
Areas of interest during lockdown. Distribution of page views (above, **a**) and session duration (below, **b**) when looking at the different areas of interest after the beginning of the lockdown.

The results of the entire period investigated are summarized in Table 3. A significant increase in the share of page views of intimate surgery topics and a significant decrease in body contouring topics were demonstrated. No further significant differences were discerned. Figure 4 displays the distribution of page views (a) and session duration (b) following the beginning of the lockdown.

**Table 3:** Share of page views (above) and session duration (below) of the respective areas of interest during the total investigated period prior- vs. after the beginning of the lockdown (Germany, Austria, and Switzerland).

	I	II	III	IV	V	Total
*Share of page views*						
Prior-	32.32%	25.37%	15.23%	13.45%	13.63%	100%
During-	32.37%	25.40%	14.54%	14.38%	13.32%	100%
*p*	0.638	0.710	0.002*	<0.001*	0.477	
*Share of session duration*						
Prior-	20.06%	20.01%	20.04%	19.91%	19.97%	100%
During-	20.16%	20.06%	19.88%	20.06%	19.84%	100%
*p*	0.522	0.492	0.138	0.098	0.252	

I = Hand-/foot-surgery, II = Facial surgery, III = Body contouring, IV = Intimate surgery, V = Breast surgery, *p* = *p*-value.

*Statistically significant.

## Discussion

Our results do not indicate a decline in the online relevance of plastic-surgical information during the first hard lockdown of the COVID-19 pandemic. This study highlights the importance of reliable and professionally validated information online, in particular during these exceptional circumstances. Furthermore, it was demonstrated that the pandemic led to more focused search habits and more conscious acquisition and retention of medical information.

### No decline in page views

The analysis of the number of page views did not reveal any significant differences prior to or during the first lockdown in the periods assessed. Overall, a tendency towards an increasing number of page views was observed, which in this study is considered representative of the increased relevance of plastic surgery content online. However, this tendency is not statistically significant over the entire study period. This is most likely due to fewer page views immediately after the beginning of the first lockdown (4 weeks *p* = 0.062). The beginning of the lockdown marks a decisive point in time, as from that point onwards, the entire population, whether informed about COVID-19 or not, was confronted with the disease and directly affected by governmental measures. The decreased page views, which only barely missed statistical significance, were possibly due to the increasing uncertainty amongst the population, which could have led to the relativization of “lifestyle medicine” like esthetic plastic surgery and information in this regard compared to the potentially life-threatening COVID-19 disease. However, the 8-week analysis shows that this effect was mostly seen during the first 4-week period assessed. When analyzing the 8-week period, the tendency towards decreased page views clearly missed statistical significance (*p* = 0.154).

Considering the entire study period (total), it becomes evident that the initial decrease in page views was only a temporary effect and that the increasing trend from prior to the lockdown actually continued. This is particularly relevant because the beginning of the lockdown was chosen as the key time point in this study, and the lockdown itself and thus the forced confrontation with COVID-19 were still relevant afterward. Even in the time prior to the lockdown, the virus and the associated illness were present, which once again supports the relevance of plastic-surgical information with an increasing trend in page views. A study by Dhanda et al. (2020) came to a similar conclusion; an initial drop, but also followed by an increase in search queries on Google for esthetic interventions was observed (Dhanda et al., 2020). Another study by Popp et al. (2021) investigated the caseload in plastic surgery during the pandemic and concluded that the majority of plastic surgery cases during the pandemic represent emergency and medically necessary surgeries (Popp et al., 2021). Even if our study does not differentiate between necessary and purely elective interventions, it nonetheless reflects the importance of plastic surgery and the interest in plastic surgery by the general population, regardless of the pandemic.

### Unaffected session duration by the pandemic

With regard to the length of stay on the website, there were no significant differences prior to and after the start of the lockdown. A page visit lasted about the same duration on average, regardless of whether society was in lockdown or not. Therefore, it could be inferred that the interest in the information provided continued, on the one hand, uninterrupted and, on the other hand, no significant increase in ‘accidental’ visits of the website via, for example, unspecific search queries in search engines, which were canceled after a short time to verify the ‘unsuitability’ was recorded.

### Targeted information acquisition through lower bounce rate

Rather, the significant decrease in bounce rates (*p*-value < 0.05 at any time) is an indication that a search for relevant information was carried out in a much more targeted manner. When the website and the corresponding information on the website have been found, the number of visitors who stayed on the website and did not immediately jump off again is significantly higher. This may suggest increasing conscientiousness with which the relevant information was being sought; and perhaps also hints at increased retention of such information. While Crockett (Crockett et al., 2007) and Heidekrueger (Heidekrueger et al., 2019) congruently report on the enormous significance of reality TV or Tabloid press as an information source for plastic surgery information, the observed development here leads to the presumption that the demand for scientifically and professionally validated information is increasing and consumed more consciously.

### Cross-thematic relevance of plastic surgery

The evaluation of the relative page views or length of stay with regard to the five areas of interest hand/foot surgery (I), facial surgery (II), body contouring (III), intimate surgery (IV) and breast surgery (V) showed a significant increase in page views of intimate surgery topics in the 8 weeks (*p* = 0.006) and total analysis periods (*p* < 0.001). In the 4-week analysis, there was no significant difference and in the overall analysis, this increase was at the expense of a significant decrease in body-contouring topics (*p* = 0.002). It is at least doubtful whether this development is due to the lockdown or the pandemic. Rather, the increasing significance over time is indicative of societal developments as a whole—away from taboos and towards increasing popularity and interest in intimate surgery (Hamori and Stuzin, 2018).

With regard to the session duration, there are no significant differences in the various areas of interest. Both, prior to and after the start of the lockdown, the session duration per area of interest was almost exactly equally divided (20 ± 0.16%). This shows that individual areas of interest did not change in terms of the need for information. Information about non-esthetic fields, such as hand surgery, was studied for as long as information about more elective and mainly esthetic areas such as facial surgery. The population’s interest in esthetic surgery was not negatively impacted by the pandemic. However, due to government regulations and recommendations of specialist societies, esthetic surgery and highly elective interventions could not be performed during the high-incidence stages of the pandemic (Giunta et al., 2020; Ozturk et al., 2020). Interestingly, some plastic surgeons unanimously reported that the demand for esthetic and/or facial interventions had increased dramatically after the end of the lockdown, as has been reported in various media (MarketWatch; The Guardian; The Washington Post). This might be due to a constant confrontation with the appearance of one’s own face in various video calls (Rice et al., 2020), and/or due to the possibility of covering the operated area during the convalescence within the scope of the mask requirements. This could explain the ongoing high demand for information even during the lockdown; at the time of this study, however, it was not possible to predict that the pandemic would last throughout the entire year of 2020 and after several more lockdowns and openings, now, in April 2021, the pandemic is far from over. Therefore, the situation may have changed drastically after our investigation period and further studies are necessary to assess the long-term effects of lockdowns on interest in plastic surgery as reflected by online search behavior.

According to the authors‘ information, this is the first study to investigate society’s online behavior during a pandemic using data from a platform with verified medical content.

### Limitations and outlook

One limitation of this study is the selection of the analysis period. While the period had to be chosen long enough to have any relevance at all (one or two weeks before or after the start of the lockdown, a punctual statement, would very likely have had no relevance), it also had to be chosen short enough to not be flooded by ‘every day’s situation’. Therefore, we elected the 4-week vs. the 8-week window. In principle, it is of course possible that other results could have been reported in 2-week windows, but in our opinion without any significant informative value. The fact that no significant differences in terms of page views and length of stay could be observed even when viewed over a 4-week period supports the thesis that online behavior was not significantly influenced by the pandemic, which had peaked in an initial lockdown at that time. The interest in topics related to plastic surgery remained constant. In this context, it should be noted once again that the duration of the pandemic was not and cannot be predicted at any point in time; therefore, this investigation covers a finite period. This excerpt could be given even more informative value by performing further investigations after the COVID-19 pandemic has subsided. One major limitation in our study is the aspect that the search behavior is influenced by many factors; all these individual and personal factors, i.e. mood, financial situation, level of boredom, and time at hand, have not been acquired as data points nor quantified. Yet, we believe our study to have yielded representative results of the German-speaking population. Another limitation is the market reach of MOOCI GmbH, which is still in the initial phase of its growth, possibly limits the range and leading to likely selection bias. This also limits the informative value with regard to a representative online population as only data from Germany, Austria, and Switzerland was considered. Future studies could be performed analyzing the traffic of, for example, the website of the American Society of Plastic Surgeons (ASPS), which provides information about procedures, similar to Mooci, in the English language. Google Analytics as a tool also has its limitations: the session duration is a factor that is not measured to the second but requires further interaction. The bounce rate can also be distorted due to a lack of interactions. This could lead to overinterpretation of our results. The exact functions and their weaknesses can be found on the Google Analytics support page (Google Analytics Help).

Subsequently, further studies should assess data over the entire course of the pandemic, also emphasizing the differentiation between esthetic and non-elective interventions. Such information could yield a better understanding of the public perception of plastic surgery and promote the distribution of reputable and reliable information about plastic surgery online.

## Conclusion

Ultimately, we were unable to completely refute or confirm our initial thesis that the pandemic had an impact on online search behavior. There was a tendency for fewer page views immediately after the start of the first lockdown with consistent session durations. However, this difference was not statistically significant and was quickly equilibrated by the website’s overall increase in page views. We were able to show a statistically significant decrease in bounce rate after the start of the lockdown, professionally curated information about plastic surgery was consequently more consciously perceived. Interest in the various topics remained unaffected, however. The provision of reliable information regarding plastic-surgical interventions continues to be a widely accepted and effective means of supporting and, if necessary, initiating successful doctor–patient interaction, in particular during a pandemic. Our findings underline the importance of readily available professional online information about plastic surgery procedures and confirm the relevance of plastic surgery independent of a worldwide crisis.

## Data Availability

All data generated or analyzed during this study are included in this published article.
